# Intensive Care Units of a Tertiary Care Hospital: A Detailed Study of Patient Symptomatology

**DOI:** 10.7759/cureus.3988

**Published:** 2019-01-30

**Authors:** Shehzeen F Memon, Anosh A Khan, Sheema Mustafa, Muznah T Farooqui, Bareerah S Khan, Sumeen Jalees

**Affiliations:** 1 Internal Medicine, Dow University of Health Sciences, Karachi, PAK; 2 Orthopaedics, Dow University of Health Sciences, Karachi, PAK

**Keywords:** nosocomial infections, intensive care units, neuropsychiatric, gastrointestinal, respiratory

## Abstract

Introduction

Nosocomial infection (NI) is a factor of considerable significance in determining the morbidity and mortality of patients admitted to the intensive care units (ICUs). Our aim was to study the frequencies of various symptoms that might emerge due to nosocomial infection (NI) among ICU patients.

Materials and methods

This was a cross-sectional study conducted in intensive care units of General Medicine, General Surgery and Paediatric wards in Ruth M. Pfau Civil Hospital Karachi, Pakistan, using well-structured questionnaire created after a thorough literature study. Patients who had an ICU admission for more than five days but less than two weeks were set as the inclusion criteria. The remaining patients were excluded from the study.

Results

Among the patients who developed gastrointestinal disturbances while in the intensive units, loss of appetite had the highest ratio in the paediatric ICU, whereas vomiting was most prevalent in the surgical ICU and weight loss was the most recurring symptom in the medical ICU. With regard to the respiratory and cardiovascular symptoms, dyspnea stood out in medicine ICU while fatigue was the most evident symptom amidst the paediatrics and surgical ICU patients. Insomnia was the most common neuropsychiatric symptom in the surgical and paediatric ICUs. Insomnia also paralleled tremors frequently in the medical ICU.

Conclusion

Infections in patients under intensive care in a tertiary care setup are not restricted to a specific type but present variously, as indicated by the type of symptoms the patients develop during their stay.

## Introduction

Intensive care units (ICU) are part and parcel of healthcare setups worldwide. They deal with both postoperative care and the consequences of an extensive hospital stay. Healthcare-acquired infection, commonly called nosocomial infection (NI), is defined by the Centre for Disease Control and Prevention (CDC) as a localized or systemic condition developing due to an adverse reaction to the presence of an infectious agent(s) or toxin(s) without any evidence that the infection was present at the time of admission to the acute care setting [1]. Due to its high morbidity and mortality rate worldwide, it is considered to be an important health problem all around the world [2] and has additional prominence in third-world countries where the infrastructure does not support healthy coping mechanisms and patients are frequently faced with infections that appear during their stay in ICUs.

Occasionally, patients who are admitted to ICUs already have an underlying disease and impaired host defenses, which puts them at higher risks of developing nosocomial infections [3]. Certain other factors that contribute to the development of ICU infections amount to different invasive and monitoring procedures, instrumental use and persistent hospital stay [3]. It has been reported that the incidence of nosocomial infections in ICUs is about two to five times higher than in the general in-patient hospital population [1], further reaffirming the fact that these infections increase the hospital stay, require extensive treatment and eventually add up to the cost of patient care [4], which in a tertiary setup in a developing country like Pakistan is again a special concern.

Tertiary setups in our area are seldom equipped with health protocols to deal with the general in-patient population, let alone patients treated in ICUs, highlighting the necessity of quantifying the consequences of ICU stay. Literature has been published from Pakistan [5-6] on infections developing in the ICUs, but details of the symptomatology of the patients have not been discussed in either. Our study intends to determine the symptoms that the patients of ICUs of Paediatrics, General Surgery and General Medicine wards develop. Determination of the risk factors for developing infections in ICU patients is a necessary precaution to take in order to prevent penalties and after-effects which can be as atrocious and horrific as death, and which affect the general population size of an already underprivileged, less-resourced country and include the additional work needed to deal with the emotional trauma that the families of such patients go through.

## Materials and methods

The following project is a cross-sectional study conducted in ICUs of General Medicine, General Surgery and Paediatric wards in a renowned tertiary care hospital, namely Ruth M.Pfau Civil Hospital, in the metropolitan city of Karachi, Pakistan, using a well-structured questionnaire created after a thorough literature study. All conscious patients, irrespective of age and gender, with an admission of five days (72 hours) minimum in respective ICUs were included in the study. Comatose, verbally impaired patients and patients staying for less than 72 hours or more than two weeks met our exclusion criteria because a longer stay could result in patients getting better after treatment with medication to relieve their ICU symptoms. For those patients who were neither of the above categories but were too exhausted or unwell to respond, full consent was taken to let their attendants elaborate the symptoms which the patients complained of developing.

The questionnaire comprised two parts, the first one discussing the demographics necessary for the patient's registration followed by the documentation of their vitals taken within the recent two hours, including blood pressure, pulse, temperature and respiratory rate noted both from the patient records and data and clinically evaluated by us for reassurance. Also, the devices and instruments used on them were registered. The second segment comprised symptoms that patients developed during their stay arranged in the form of a checklist separately for the ICU of each ward. These symptoms were further organized into three categories of gastrointestinal, respiratory and neurological, combined with psychiatric symptoms. A total of 300 patients took part in our study, 100 from each of the three categories of the ICU. The questionnaires that were returned unfilled were discarded. The final data was entered into the SPSS (Statistical Package for the Social Sciences) software version 22 (IBM, NY, USA) and analyzed by taking out frequencies of each symptom in all the selected ICUs using descriptive statistical test for frequencies.

## Results

The results of this study showed that amongst the patients who developed gastrointestinal disturbances, loss of appetite (*n *= 74) was a common occurrence, being in the highest ratio in the paediatric ICU (*n *= 37), whereas jaundice (*n *= 25) was the least developed symptom reported overall with most cases found in the surgical ICU (*n *= 10). Vomiting was also a commonly developed symptom in the surgical ICU (*n *= 22), so was weight loss, presenting in 24 patients amongst a number of 59. Further details are given in Table 1.

**Table 1:** Gastrointestinal symptoms occurring in ICUs ICU: intensive care unit

Gastrointestinal symptoms	Medical ICU patients N (%)	Surgical ICU patients N (%)	Paediatric ICU patients N (%)	Total patients who developed symptoms (N)
Vomiting	12 (25.5%)	22 (46.8%)	13 (27.65%)	47
Constipation	10 (38.4%)	13 (50%)	3 (11.5%)	26
Weight loss	15 (25.4%)	24 (40.6%)	20 (33.8%)	59
Jaundice	5 (20%)	10 (40%)	10 (40%)	25
Loss of appetite	10 (13.5%)	27 (36.5%)	37 (50%)	74

The results in Table 2 also painted a clear picture of the respiratory and cardiovascular derangements that patients developed in the form of dyspnea (shortness of breath), cough, syncope and palpitations. Most patients complained of dyspnea (*n *= 37), which occurred in the highest proportion in the medical ICUs, followed by cough (*n *= 29). Fatigue was a substantial occurrence in surgical (*n *= 21) and paediatric ICU (*n *= 15) patients, even though fatigue might have been mistaken for irritability and general sleepiness in children in the latter case.

**Table 2:** Respiratory and cardiovascular symptoms occurring in ICUs ICU: intensive care unit

Respiratory and cardiovascular symptoms	Medical ICU patients N (%)	Surgical ICU patients N (%)	Paediatric ICU patients N (%)	Total patients who developed symptoms (N)
Dyspnea	37 (37.7%)	26 (26.5%)	35 (35.7%)	98
Cough	29 (43.9%)	13 (19.6%)	24 (36.3%)	66
Syncope	6 (42.8%)	4 (28.5%)	4 (28.5%)	14
Palpitations	9 (42.8%)	9 (42.8%)	3 (14.2%)	21
Fatigue	11 (23.4%)	21 (44.6%)	15 (31.9%)	47

We also evaluated our patients on neurological and psychiatric grounds. Symptoms such as anxiety, insomnia and tremors were primarily considered. The results showed that insomnia (*n *= 48) was reported the most as a whole, being commonest in the surgical ICU (*n *= 25), followed by anxiety (*n *= 19) as compared to other ICUs. Insomnia was a frequently reported symptom in paediatric patients (*n *= 13), whereas tremors were reported equally from the medicine and paediatric ICU, presenting as the least occurring symptom overall (*n *= 24). Further details have been displayed in Table 3.

**Table 3:** Neuropsychiatric symptoms occurring in ICUs ICU: intensive care unit

Neuropsychiatric symptoms	Medical ICU patients N (%)	Surgical ICU patients N (%)	Paediatric ICU patients N (%)	Total patients who developed symptoms (N)
Anxiety	3 (11.1%)	19 (70.3%)	5 (18.5%)	27
Insomnia	10 (20.8%)	25 (52%)	13 (27%)	48
Tremors	10 (41.6%)	4 (16.6%)	10 (41.6%)	24

Conclusively, the derangements faced by patients during their ICU stay, when classified into three sets, displayed that most of the patients being monitored in all the ICUs developed dyspnea in that spell, but, cough was also a major symptom developed in the patients being taken care of at medicine ICU while anxiety was a common matter of concern in surgical ICU patients. Meanwhile, appetite loss was the highlight of symptoms developing in paediatric ICU patients.

## Discussion

Critical patients bound to stay in ICUs for extensive durations develop certain significant, characteristic derangements of metabolism and of neuroendocrine, neuropsychiatric and immunologic functions in their bodies [7]. Our study intended to evaluate these imbalances by observing the medical and physical symptoms patients developed whilst their stay in the ICUs. Compared to first-world countries, the ICUs of low-income, third-world countries may have to cope with patients suffering from severe illnesses coupled with the lack of resources and expertise needed to control nosocomial infections [8]. Pakistan, being one of them, lacks research on the subject of patient symptomatology in Intensive care units. 

A spectrum of gastric symptoms was seen in our patients admitted to the ICUs (Table 1), of which, appetite loss stood out. Nematy et al. [9] worked on changes in appetite in 129 ICU patients and found a significant alteration in the nutritional habits of practically all of them. This suggests that appetite loss is a major problem faced by patients in the ICU. Studies have proposed that the involvement of gastrointestinal hormones like glucagon-like peptide (GLP-1) plays a major part in this alteration [10]. Loss of appetite may develop during hospital stays either as a consequence of infection or due to medications and treatments; it may pre-exist as a primary condition owing to depression, social isolation and advanced age. In our tertiary care arrangement, the unhygienic preparation and mishandling of food after it is brought to the hospital for patients can also be a noteworthy contributor to patients losing their appetite, as a majority (*n *= 37) of patients from the total were children in the paediatric ICU who could fall ill due to contamination earlier than adults. A considerable number of studies found more than half of the subjects registering vomiting as a primary gastrointestinal complaint [10-12]. However, patients in our study reported vomiting in much lesser frequency possibly due to the avoidance of food during their stay. Factors like nasogastric aspiration and enteral feeding which are commonly administered in ICU patients may have contributed to the development of this symptom. Mishandling of feeding devices and contamination again plays a role in tertiary care setups like ours. 

Dyspnea (*n *= 98) and cough (*n *= 66) are also frequently reported symptoms in our study, as seen in Table 2, mainly in the medicine ICU, and in all age groups, possibly owing to the fact that respiratory infections are prevalent in our setup where ICUs are not completely packed and the flow of people is not controlled. A very common health issue in Pakistan is tuberculosis. The organisms of this disease are latent in a majority of the population, which includes doctors, nursing staff and medical students, all of whom have access to the ICUs, further deteriorating the previously weak immune system of the ill. These symptoms may also develop in the patients due to them habitually acquiring nosocomial pneumonia in our systems. Likewise, Puntillo et al. [13] found dyspnea as the most disturbing symptom in a study of patient symptomatology in ICUs. However, their study focused more on patients admitted due to terminal diseases like lung cancer while ours focused both on the acutely ill and the chronically ill. 

Neuropsychiatric symptoms were equally explored because a psychiatric evaluation of an ill patient is an essential part of their work-up; hence, missing out on the specifics of patient anxiety and insomnia hinder their betterment and eventual relief from the ICU environment. Anxiety was found to be a major issue in the General Surgery ICU shown in Table 3, possibly because of the postoperative state of mind of the patients. Adding to this, wound infection and failure rates of the operation have contributed to some of the reporting of these symptoms. This was similar to the study of Anne Scott [14] who worked on elements of anxiety in surgical ICU patients and concluded that a majority developed anxiety before the surgery because of ‘fear of the unknown' and fear of postoperative complications such as pain, which made them anxious during their stay. Lack of sleep was also protested by our patients, and so, the possibility of insomnia being an irritant in hospital stay is not to be overlooked. Cooper et al. [15] reported that none of their mechanically ventilated patients displayed a normal sleep pattern. Triggers for insomnia in our patients who reported similarly might have been the constant noise and flow of people in and out of the ICU, patients' own preoccupations about their illnesses and most importantly their pain. 

Mechanically ventilated patients, for whatever reasons, markedly swift from the normal both physically and mentally and evaluating the reasons for this change can help the healthcare facility better assess their status, expect the outcomes and help in early recovery of these patients as not only do the sick but the families of the sick also suffer equally in the whole course.

## Conclusions

In a low-income, third-world country like Pakistan, hospital management should be extra vigilant to prevent nosocomial infections as the prevailing symptoms aggravate the fragile condition of the patients admitted in ICUs, prolong hospital stays, increase mortality risk and add more weight to the pockets of the patients and their family. A set of updated hygiene and proper patient-attendant contact rules should be set to avoid nosocomial infection as much as possible.
